# Association of the Lactase Persistence Haplotype Block With Disease Risk in Populations of European Descent

**DOI:** 10.3389/fgene.2020.558762

**Published:** 2020-10-29

**Authors:** Shannon E. K. Joslin, Blythe P. Durbin-Johnson, Monica Britton, Matthew L. Settles, Ian Korf, Danielle G. Lemay

**Affiliations:** ^1^Department of Animal Science, UC Davis, Davis, CA, United States; ^2^UC Davis Genome Center, Davis, CA, United States; ^3^Department of Public Health Sciences, UC Davis School of Medicine, Davis, CA, United States; ^4^Department of Molecular and Cellular Biology, UC Davis, Davis, CA, United States; ^5^USDA ARS Western Human Nutrition Research Center, Davis, CA, United States; ^6^Department of Nutrition, UC Davis, Davis, CA, United States

**Keywords:** human evolution, population genetics, diet, physiological traits, phenotype, selective sweep, lactose, lactose tolerance

## Abstract

Among people of European descent, the ability to digest lactose into adulthood arose via strong positive selection of a highly advantageous allele encompassing the lactase gene. Lactose-tolerant and intolerant individuals may have different disease risks due to the shared genetics of their haplotype block. Therefore, the overall objective of the study was to assess the genetic association of the lactase persistence haplotype to disease risk. Using data from the 1000Genomes project, we estimated the size of the lactase persistence haplotype block to be 1.9 Mbp containing up to 9 protein-coding genes and a microRNA. Based on the function of the genes and microRNA, we studied health phenotypes likely to be impacted by the lactase persistence allele: prostate cancer status, cardiovascular disease status, and bone mineral density. We used summary statistics from large genome-wide metanalyses—32,965 bone mineral density, 140,306 prostate cancer and 184,305 coronary artery disease subjects—to evaluate whether the lactase persistence allele was associated with these disease phenotypes. Despite the fact that previous work demonstrated that the lactase persistence haplotype block harbors increased deleterious mutations, these results suggest little effect on the studied disease phenotypes.

## Introduction

Lactose is the main carbohydrate found in milk. The enzyme lactase, encoded by the *LCT* gene, allows for the breakdown of lactose in infant mammals. Various human populations continue to express *LCT* post weaning and can digest lactose into adulthood, a trait known as lactase persistence (LP) (Swallow, 2003). The rs4988235 (−13910 C > T) transition variant, or LP allele, in the promoter of the *LCT* gene allows for LP in populations of European descent. The allele frequency of this advantageous mutation rapidly rose in groups with milk and dairy production and consumption relatively recently, as seen by the signature of a relatively large haplotype block surrounding the LP allele (Bersaglieri et al., 2004; Itan et al., 2009; Ségurel and Bon, 2017). Therefore, lactose tolerant and intolerant individuals’ genetic backgrounds differ in the alleles surrounding the LP allele. Positive selection for the trait of LP can hold slightly deleterious alleles that are in linkage disequilibrium (LD) with the LP allele at a higher frequency than expected under balancing selection alone (Smith and Haigh, 1974; Fay and Wu, 2000; Chun and Fay, 2011; Cutter and Payseur, 2013). Prior work by Chun and Fay (2011) found European samples harbored multiple deleterious or neutral non-synonymous SNPs within the *LCT* gene and two other genes in the surrounding the region. However, it is unclear whether mutations found within the LP haplotype block give rise to unfavorable phenotypes. Determining the differential risk of disease based on individual genetic backgrounds with the indirect phenotype of lactase persistence may help resolve contrasting epidemiological findings and improve public health. Therefore, the objective this study was to determine the size of the LP haplotype block and its impact on disease risk in humans with and without the LP allele.

## Materials and Methods

### Data Sets

#### Genotype

The 1000 Genomes Phase 3 datasets were accessed on March 15, 2016 from the organization’s data portal^1^. Unrelated individuals from the 1000 Genomes ethnic classifications of Northern and Western European Ancestry (CEU), Finish (FIN), English or Scottish (GBR), Iberian (IBS) or Italian (TSI) were used to define the lactase persistence haplotype block.

#### Osteoporosis

Data used in our osteoporosis analyses were downloaded from the 2015 data release of the GEFOS single variant bone mineral density (BMD) meta-analysis dataset comprised of individuals of European ancestry (Zheng et al., 2015). We accessed the genome-wide meta-analysis summary statistics from the genome-wide association meta-analysis (GWAMA) of femoral neck BMD, forearm BMD and lumbar spine BMD using 32,965 individuals in the publicly accessible GEFOS database^2^ in July 2018. To date, the GEFOS study is the largest publicly accessible femoral neck, lumbar spine and forearm bone mineral density genome-wide meta-analysis dataset.

#### Prostate Cancer

In order to carry out our prostate cancer analyses we accessed summary association statistics data from the publicly accessible Prostate Cancer Association Group to Investigate Cancer Associated Alterations in the Genome (PRACTICAL) consortium Oncoarray database^3^ in July 2018. Genome-wide meta-analysis statistics of prostate cancer from 79,194 prostate cancer cases and 61,112 controls of European ancestry were made publicly available in 2018 from the PRACTICAL dataset and were subsequently used in our analyses (Schumacher et al., 2018).

#### Coronary Artery Disease

Meta-analysis summary data for individuals with coronary artery disease were accessed from the CARDIoGRAMplusC4D 1000 Genomes-based GWAS dataset^4^ in October 2017. The dataset is a meta-analysis of GWAS studies of mainly European, South Asian, and East Asian descent imputed using the 1000 Genomes phase 1 v3 training set with 38 million variants and was made publicly available in 2015. The study interrogated 9.4 million variants and involved 60,801 CAD cases and 123,504 controls (Nikpay et al., 2015). To date, this dataset is the most comprehensive GWAS of coronary artery disease in populations of European descent.

### Analysis

#### Characterizing Lactase Persistence LD

We determined the pattern of LD with the lactase persistence T allele at rs4988235 in populations of European descent from the 1000 Genomes Phase 3 datasets. Unrelated subjects of CEU, FIN, GBR, IBS or TSI ethnicity were considered for the purpose of our analysis. Downloaded data were converted from vcf to PLINK formatted ped/map files using the fcgene software (Roshyara and Scholz, 2014). Genome-wide pairwise associations with marker rs4988235 (2:136608646_G_A) were calculated in PLINK (Purcell et al., 2007). Pairwise calculations were conducted using 1,000,000 markers and 1,000,000,000 base pair windows. Thus, the upper limit of distance or pairwise comparisons for the marker of interest shall not exceed more than 1 M markers nor 100 Mb. LD was measured using *r*^2^ and calculated with the equation: $r^{2}\left(p_{a},p_{b},p_{ab}\right)=\frac{{\left(p_{ab}-p_{a}p_{b}\right)}^{2}}{p_{a}\left(1-p_{a}\right)p_{b}\left(1-p_{b}\right)}$, where *p*_*a*_, *p*_*b*_ and *p*_*ab*_ denote the frequencies of the a and b alleles and the ab haplotype, respectively. A SNP with *r*^2^ > 0.2 was considered in LD with rs4988235 for the purpose of this study. The upper and lower boundaries of European LP haplotype block were determined by selecting the most distant SNPs with an *r*^2^ > 0.2 with rs4988235.

#### Omnibus Effect

To test if SNPs in high LD with the LP SNP are also associated with a disease of interest, class-level genetic association tests (GenCAT) were performed for individual phenotypes within each disease using the GenCAT package in R (Qian et al., 2016). These tests use a user defined “class” of SNPs, in this case SNPs in high LD (*r*^2^ > 0.2) with the LP SNP, to see if either the number of SNPs in the class or correlations of the SNPs in the class are statistically meaningful when compared to the SNPs associated with a disease of interest. Log odds ratios for all effect alleles were aligned to be in phase with the lactase persistence haplotype. SNPs in high LD (*r*^2^ > 0.2) were considered a class and tested to determine if class had an effect on all phenotypes for each disease. A total of five phenotypes corresponding to three different diseases were evaluated for a class effect of LP haplotype block on the phenotype of interest (Table 1). We investigated class associations of the lactase persistence haplotype block on the following phenotypes: femoral neck bone mineral density, forearm bone mineral density, lumbar spine bone mineral density, individuals with prostate cancer, and individuals with coronary artery disease.

**TABLE 1:** Disease phenotypes, measurements and corresponding test statistics.

Phenotype	Measure	Fisher’s exact test *P*-value	Wilcoxon *P*-value, test on two-sided *P*-values	Wilcoxon *P*-value, test on upper *P*-values	Wilcoxon *P*-value, test on lower *P*-values	Mean *B*eta for SNPs in *P*hase
Prostate cancer	Affected	1	0.001393	1	1.611e-7	1.021982
Coronary artery disease	Affected	1	2.2e-16	2.545e-11	1	0.995843
BMD	Femoral neck	1	0.03217	1	2.2e-16	1.006456
BMD	Lumbar spine	1	1	1	2.2e-16	1.005034
BMD	Forearm	1	2.2e-16	1	2.2e-16	1.009849

#### Enrichment Analyses

To determine if disease-associated markers were enriched in the lactase persistence haplotype, the distribution of *p*-values associated with SNPs in linkage disequilibrium and equilibrium were compared. A Fisher’s Exact Test was used to test if the proportion of markers with an adjusted *p*-value less than 0.05 (Bonferroni correction) in each phenotype’s meta-analysis results were higher among SNPs in high LD (r^2^ > 0.2) with the lactase persistence SNP than expected under homogeneity. Wilcoxon rank sum tests were used to test if markers in high LD (*r*^2^ > 0.2) with the lactase persistence marker (rs4988235) had significantly smaller *p*-values in a meta-analysis than those not in high LD. For each meta-analysis, this test was performed on three different sets of *p*-values:

(1) Two-sided *p*-value (i.e., the raw *p*-value provided in the disease phenotype meta-analysis results), to test if the log odds ratio beta does not equal zero, i.e., an effect of a SNP on the case status in either direction. A significant enrichment test based on these *p*-values means that the SNPs most associated with case status (e.g., CAD, prostate cancer), or BMD (regardless of direction) are enriched for SNPs in LD with the lactase persistence SNP.
(2) One-sided, upper, *p*-value to test if beta is greater than zero (derived from the Z statistic in the disease phenotype meta-analysis table), i.e., if a SNP is associated with a higher odds of case status or higher BMD. A significant enrichment test based on these *p*-values means that the SNPs most associated with higher odds of case status (e.g., CAD, prostate cancer), or higher BMD, are enriched for SNPs in LD with the lactase persistence SNP.
(3) One-sided, lower, *p*-value to test if beta is less than zero (derived from the Z statistic in the disease phenotype meta-analysis table), i.e., if a SNP is associated with a lower odds of case status or lower BMD. A significant enrichment test based on these *p*-values means that the SNPs most associated with lower odds of case status (e.g., CAD, prostate cancer), or lower BMD, are enriched for SNPs in LD with the lactase persistence SNP.

#### Software

The software versions used in this paper are R versions 3.3.1, 3.3.2, and 3.4.0, fcgene-1.0.7 (Roshyara and Scholz, 2014), METAL version 2011-3-25 (Willer et al., 2010), and PLINK v1.90p 64-bit (Purcell et al., 2007). Analyses were conducted using the following R packages: GenCAT version 1.0.3 (Qian et al., 2016), SNPRelate version 1.8.0 (Zheng et al., 2012), snpStats version (Solé et al., 2006).

## Results

### Patterns of Linkage Disequilibrium

**FIGURE 1:**
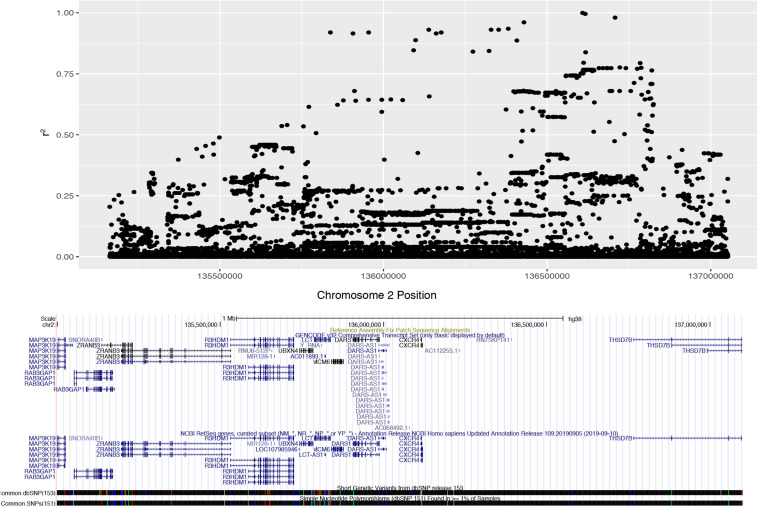
Expanded Manhattan plot of lactase persistence haplotype block on chromosome 2 encompassing nine genes and one microRNA found in the human GRCh38 assembly. SNPs associated (*r*^2^ > 0.2) with rs4988235 (chr2:135,851,076) span from rs3791298 (chr2:135,163,759) to rs6706934 (chr2:137,052,317).

We determined the pattern of LD with rs4988235 in populations of European descent from the 1000 Genomes Phase 3 datasets. Unrelated subjects of CEU, FIN, GBR, IBS or TSI ethnicity were considered for the purpose of our analysis. These individuals (*n* = 407) had a minor allele frequency (MAF) of 0.497852 for the lactase non-persistent (ancestral) C allele at rs4988235 (2:136608646_G_A). The European lactase persistence haplotype block (*r*^2^ > 0.2) was 1.89 Mb, spanned from rs3791298 (135,163,759) to rs6706934 (137,052,317) on chromosome 2 and contained 1,187 SNPs in high LD with rs4988235 (Figure 1, Supplementary Figures S2, S3). This linkage block of contains at least nine protein-coding genes and one micro-RNA. The protein-coding genes include RAB3GAP1, ZRANB3, R3HDMI, UBXN4, *LCT*, MCM6, DARS, CXCR4, THS7B, and possibly MAP3K19. The microRNA is MIR128-1. These genes and their known functions are listed in Supplementary Table S1. The microRNA, MIR-128-1, and two genes—CXCR4 and THSD7B—in the *LCT* locus all have putative roles in prostate cancer (Supplementary Table S2). We also manually reviewed the 100 SNPs most highly correlated with the lactase persistence SNP for known health associations in the literature. Numerous SNPs in the *LCT* locus were associated with total cholesterol or cardiovascular disease (Supplementary Table S3). Thus, the health phenotypes of prostate cancer and cardiovascular disease where chosen for further study. Bone mineral density phenotypes were also chosen for study due to the association of milk consumption with fracture risk in a cohort of people of European descent in which there was no assessment of genetics (Michaëlsson et al., 2014).

### Health Phenotypes

For the health phenotypes of interest, we collected publicly available summary statistics from genome-wide meta-analyses and applied two types of statistical tests. For the first type of test—a class-level genetic association test (GenCAT)—used SNP-level meta-analysis test statistics across the class of all SNPs in high LD with the LP SNP (*r*^2^ > 0.2) to determine whether that class of SNPs (the *LCT* locus) was statistically meaningful, given the size of the class and its unique correlation structure (Qian et al., 2016). The second type of statistical tests were enrichment tests to determine whether the SNPs in the *LCT* locus were enriched for phenotype-specific significance (based on SNP-level meta-analysis test statistics) relative to SNPs not in the locus (see Methods). In addition to the statistical tests, the mean beta for each phenotype was computed across all SNPs in the *LCT* locus as measure of the association of that phenotype with the *LCT* locus.

#### Prostate Cancer

**FIGURE 2:**
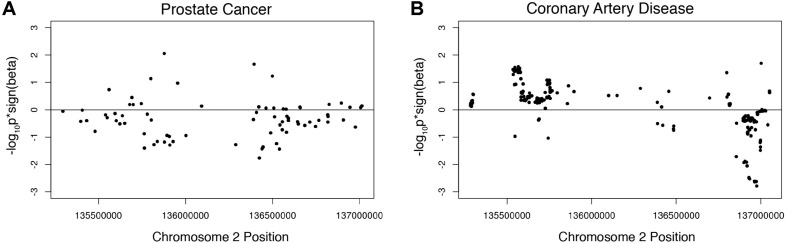
Wilcoxon rank sum tests showing beta signed *p*-values for **(A)** prostate cancer associated SNPs in high LD with the lactase persistence SNP and **(B)** CAD associated SNPs in high LD with the lactase persistence SNP.

We investigated the association of the European lactase persistence haplotype block with prostate cancer risk using genome-wide meta-analysis statistics of prostate cancer from 79,194 prostate cancer cases and 61,112 controls of the PRATICAL Consortium Oncoarray database (Schumacher et al., 2018). A Manhattan plot of the LP haplotype block in the context of chromosome 2 for the GWAS study of prostate cancer shows a SNP above -log10(P) > 3, but no SNPs rise to the level of significance of a standard GWAS (Supplementary Figure S3). The odds ratio of the LP SNP itself for prostate cancer risk was −0.01518. An omnibus analysis via a class-based association test (GenCAT) was conducted to determine if the lactase persistence haplotype block was associated with prostate cancer risk. The omnibus analysis revealed no significant association of markers in high LD with the LP SNP (*p* = 1, Supplementary Table S4). Next, enrichment analyses were conducted to determine if the distribution of *p*-values associated with SNPs in linkage disequilibrium with the LP allele differed from those associated with SNPs in equilibrium with the LP allele. Fisher’s exact test revealed no significant difference in the proportion of prostate cancer-associated SNPs being higher among SNPs in high LD with the lactase persistence SNP than would be expected under homogeneity. The Wilcoxon rank sum tests revealed that *p*-values from GWAS were smaller for SNPs in LD than for those not in LD, regardless of the sign of beta, *p* = 0.001393). A one-sided Wilcoxon rank sum test revealed SNPs in high LD with the lactase persistence SNP had smaller *p*-values for one-sided tests of beta < 0, compared to SNPs not in high LD with lactase persistence (*p* = 1.611e-7, Figure 2A) suggesting a possible negative association of the *LCT* locus with prostate cancer disease relative to SNPs not in the locus. However, the mean beta for all SNPs in LD with LP was 1.02, indicating that the odds of the LP allele being associated with prostate cancer participants is not lower, but is nearly the same as association with controls. The former test (using Wilcoxon) is a comparison of the odds ratios in the *LCT* locus compared to the odds ratios outside of the *LCT* locus while the latter measurement (mean beta) is a statement about the odds ratios in the *LCT* locus taken by themselves. While the results are equivocal on whether there is a lower risk of prostate cancer with the LP allele, one can safely conclude that the LP allele does not significantly increase prostate cancer risk.

#### Coronary Artery Disease

Given the known SNPs associated with total cholesterol in the *LCT* locus (Supplementary Table S3), we next investigated the association of this locus with the risk of coronary artery disease (CAD) using summary data from CARDIoGRAMplusC4D 1000 Genomes-based GWAS dataset which included 60,801 cases and 123,504 controls (Nikpay et al., 2015). The odds ratio of the LP allele was 0.0141738 in the coronary artery disease dataset. A Manhattan plot of the LP haplotype block in the context of chromosome 2 suggests that no individual SNPs in this block are significant for CAD (Supplementary Figure S4). The omnibus analysis revealed no significant association of markers in high LD with the LP allele with coronary heart disease (*p* = 1, Supplementary Table S4). The Fisher’s exact test revealed no significant difference in the proportion of CAD case-associated SNPs being higher among SNPs in high LD with the lactase persistence SNP than would be expected under homogeneity. A one-sided Wilcoxon rank sum test revealed CAD-associated SNPs in high LD with the lactase persistence SNP had smaller *p*-values from the one-sided test that beta > 0, compared to SNPs not in high LD with lactase persistence (*p* = 2.545e-11, Figure 2B, Table 1), suggesting a possible increase in odds of coronary artery disease associated with SNPs in LD with the LP allele, relative to those not in LD. However, the mean beta for all SNPs in LD with LP was 0.99 (Table 1), suggesting that the odds of the LP allele being associated with coronary artery disease is nearly the same as with controls. Taken together, there is limited support for an effect of the LP locus on the risk of CAD.

#### Bone Mineral Density

**FIGURE 3:**
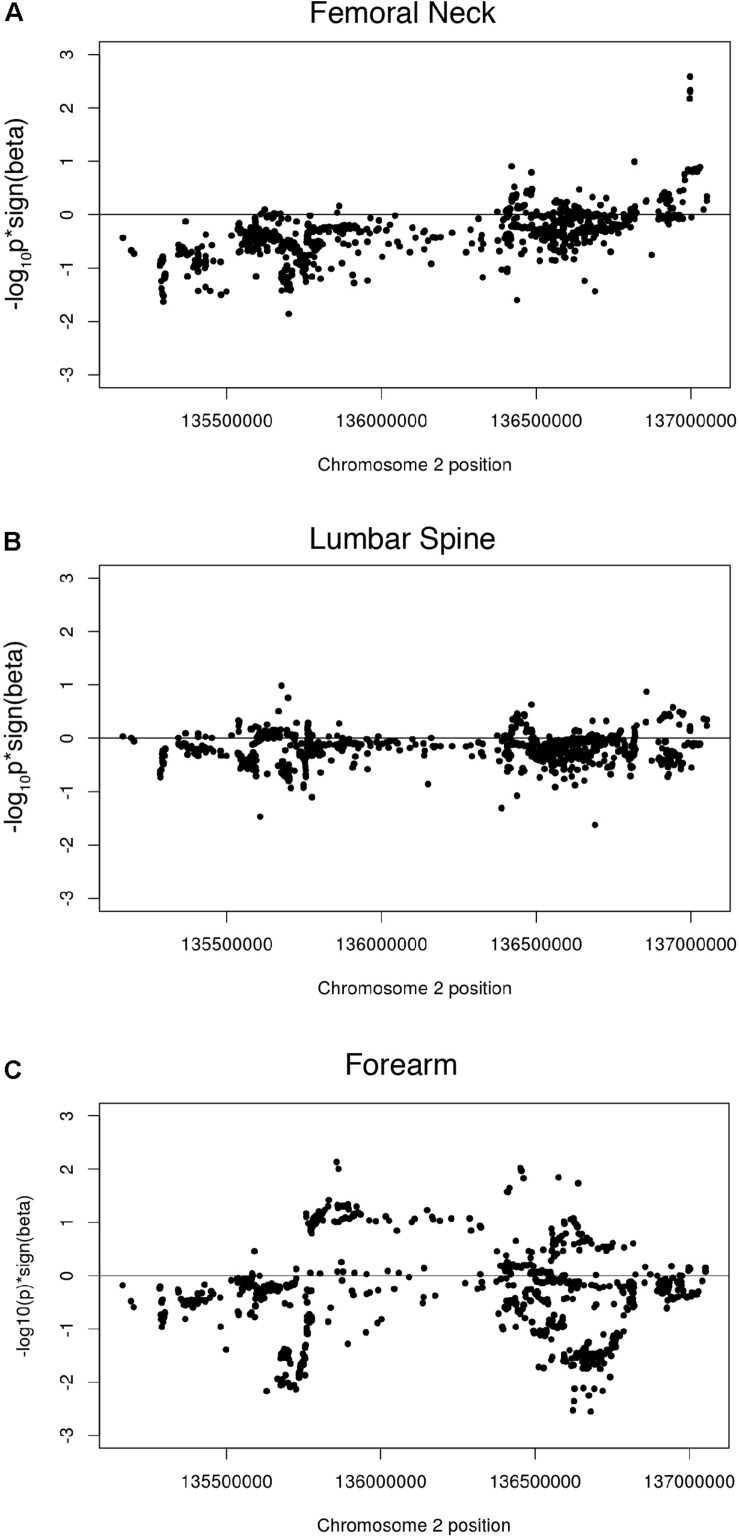
Wilcoxon rank sum tests showing beta signed *p*-values for **(A)** femoral neck BMD associated SNPs in high LD with the lactase persistence SNP; **(B)** lumbar spine BMD associated SNPs in high LD with the lactase persistence SNP and **(C)** forearm BMD associated SNPs in high LD with the lactase persistence SNP.

While most studies have shown a positive association of dairy consumption with bone mineral density, the paradoxical findings of increased fracture risk with increased milk consumption in a cohort of European descent suggested the potential for a genetic risk shared among those with the LP allele. We therefore investigated associations of the LP locus with bone mineral density (BMD), using genome-wide meta-analysis summary statistics of BMD of the femoral neck, the forearm, and the lumbar spine from 32,965 individuals in the Genetic Factors for Osteoporosis Consortium (GEFOS) consortium. Manhattan plots of the LP haplotype block in the context of chromosome 2 suggests that no individual SNPs in this block are significant for any of the three BMD traits (Supplementary Figures S5–S7). Like the other phenotypes, the GenCAT analyses revealed no significant association of markers in high LD with the LP allele for any of the BMD measurements (*p* = 1, Supplementary Table S4). A one-sided Wilcoxon rank sum test revealed femoral neck, lumbar spine and forearm associated SNPs in high LD with the lactase persistence SNP had smaller *p*-values for one-sided tests of beta < 0, compared to SNPs not in high LD with lactase persistence (*p* = 2.2e-16, Figures 3A–C, Table 1), suggesting a possible negative association of the *LCT* locus with bone mineral density relative to SNPs not in the locus. However, the mean beta for femoral neck, lumbar spine, and forearm BMD for all SNPs in phase with the LP SNP was approximately 1.01 for all three BMD sites (Table 1), suggesting little effect of the LP locus on BMD.

## Discussion

In the current study we investigated a locus in humans that encompasses the lactase gene (LCT) and shows signatures of strong, recent, positive selection with a relatively high deleterious mutation load, indictive of genetic hitchhiking. We identified the LP haplotype block in populations of European descent to be 1.9 Mb with at least nine protein-coding genes and one micro-RNA. Next, we investigated the association of the haplotype block with disease risk, prioritizing phenotypes based on known associations with SNPs in the region or with dairy consumption. Class-level (e.g., the class of all SNPs in the locus) associations were not significant for any of the phenotypes. Some of the enrichment tests were significant, but in the opposite direction of the sign of the average beta. Thus, despite recent selection for the lactase persistence allele there is little evidence that the LP haplotype block is associated with bone mineral density nor the risk of prostate cancer or coronary heart disease.

Between people who harbor the LP allele and those who don’t, there are potential differences due to both genetics (e.g., DNA hitchhiking with the LP SNP in those with the LP allele) and dairy consumption. There was no information about dairy consumption for the participants included in the current analysis. However, a recent study of healthy United States adults demonstrated that the LP genotype was only a very weak predictor of recent dairy intake as measured using 24-h recalls and was not significantly associated with overall habitual dairy intake measured using a food frequency questionnaire (Chin et al., 2019). Nevertheless, we are not able to quantify the contribution of dairy consumption to health phenotypes in the current study.

Prior studies have indicated that dairy consumption is associated with improved bone mineral density (Chan et al., 1995; Tai et al., 2015) and protective against cardiovascular disease (Alexander et al., 2016; Gholami et al., 2017; Dehghan et al., 2018), but potentially increases the risk of prostate cancer (Song et al., 2013; Aune et al., 2015). The relationship between dairy consumption and prostate cancer risk is controversial; a recent prospective investigation of 49,472 men in the United States found no association between dairy consumption and risk of prostate cancer (Preble et al., 2019). The fact that the lone microRNA in the *LCT* haplotype block has previously been shown to have a role in prostate cancer (Jin et al., 2014; Sun et al., 2015) implicated a potential for the genetics of the *LCT* locus to affect prostate cancer. However, we did not find evidence of this. MicroRNAs are highly redundant; for example, there is not a known knockout experiment of microRNAs in which their function has been shown to be essential. Thus, it is possible that deleterious mutations in or near the microRNA would have no effect on the phenotype.

Overall, across all studied phenotypes, there does not appear to be a notable effect of the LP locus on disease risk. This is surprising given the accelerated selection of the LP allele with increased potential for genetic hitchhiking and the fact that deleterious mutations have occurred in this locus at an increased rate (Chun and Fay, 2011). Our results suggest that these deleterious mutations have not given rise to unfavorable phenotypes for the studied diseases. Although it is possible that we have not studied a relevant phenotype, our manual review of the locus found SNPs or genes related to total cholesterol and prostate cancer, suggesting that cardiovascular disease and prostate cancer were the two phenotypes most likely to be affected by the genetics of the locus. Separately, the paradoxical findings of high milk consumption associated with increased fracture rate in a large cohort of European descent (Michaëlsson et al., 2014) might be explained by a genetic contribution given that dairy consumption is known to be associated with improved bone mineral density (Chan et al., 1995; Gholami et al., 2017; Dehghan et al., 2018). Thus, we covered the phenotypes most likely to be affected by the SNPs at the *LCT* locus and yet did not find an association of the locus with the phenotypes suspected to be affected. Additionally, we did not analyze other SNPs associated with LP, such as rs182549 (−22018G > A), due to having weaker associations of the derived allele and the LP phenotype (Enattah et al., 2002; Ségurel and Bon, 2017). Finally the rs4988235 T allele is not strictly predictive of the LP phenotype due to epigenetic effects (Leseva et al., 2018). Overall, our results suggest that rapid positive selection of the *LCT* locus and an increase in deleterious mutations have not translated into unfavorable disease phenotypes in humans.

## Data Availability Statement

Publicly available datasets were analyzed in this study. This data can be found here: http://ftp.1000genomes.ebi.ac.uk/vol1/ftp/technical/reference/phase2_reference_assembly_sequence/hs37d5.fa.gz; http://www.gefos.org/sites/default/files/README.txt; http://practical.icr.ac.uk/blog/?page_id=8164; www.CARDIOGRAMPLUSC4D.ORG.

## Author Contributions

SJ carried out manual reviews, contributed to the collection of the datasets, carried out bioinformatic analyses, interpreted results, and wrote the manuscript. BD-J conducted statistical analysis and interpretation. MB conducted bioinformatics analyses. MS and IK provided bioinformatics guidance. DL conceived and directed the study, coordinated the collection of the datasets, interpreted results, and wrote the manuscript. All authors contributed to study design, reviewed and edited the manuscript.

## Conflict of Interest

The authors declare that the research was conducted in the absence of any commercial or financial relationships that could be construed as a potential conflict of interest.

## Footnotes

1 ftp.1000genomes.ebi.ac.uk//vol1/ftp/technical/reference/phase2_reference_assembly_sequence/hs37d5.fa.gz

2 http://www.gefos.org/sites/default/files/README.txt

3 http://practical.icr.ac.uk/blog/?page_id=8164

4 www.CARDIOGRAMPLUSC4D.ORG
