# Supplementary material for: Association of the Lactase Persistence Haplotype Block With Disease Risk in Populations of European Descent
Source: Front Genet. 2020 Oct 29;11:558762. doi: 10.3389/fgene.2020.558762 (PMC7658388; doi:10.3389/fgene.2020.558762)
Supplement: Supplementary file 1 [file Presentation_1.pptx]

## Slide 1
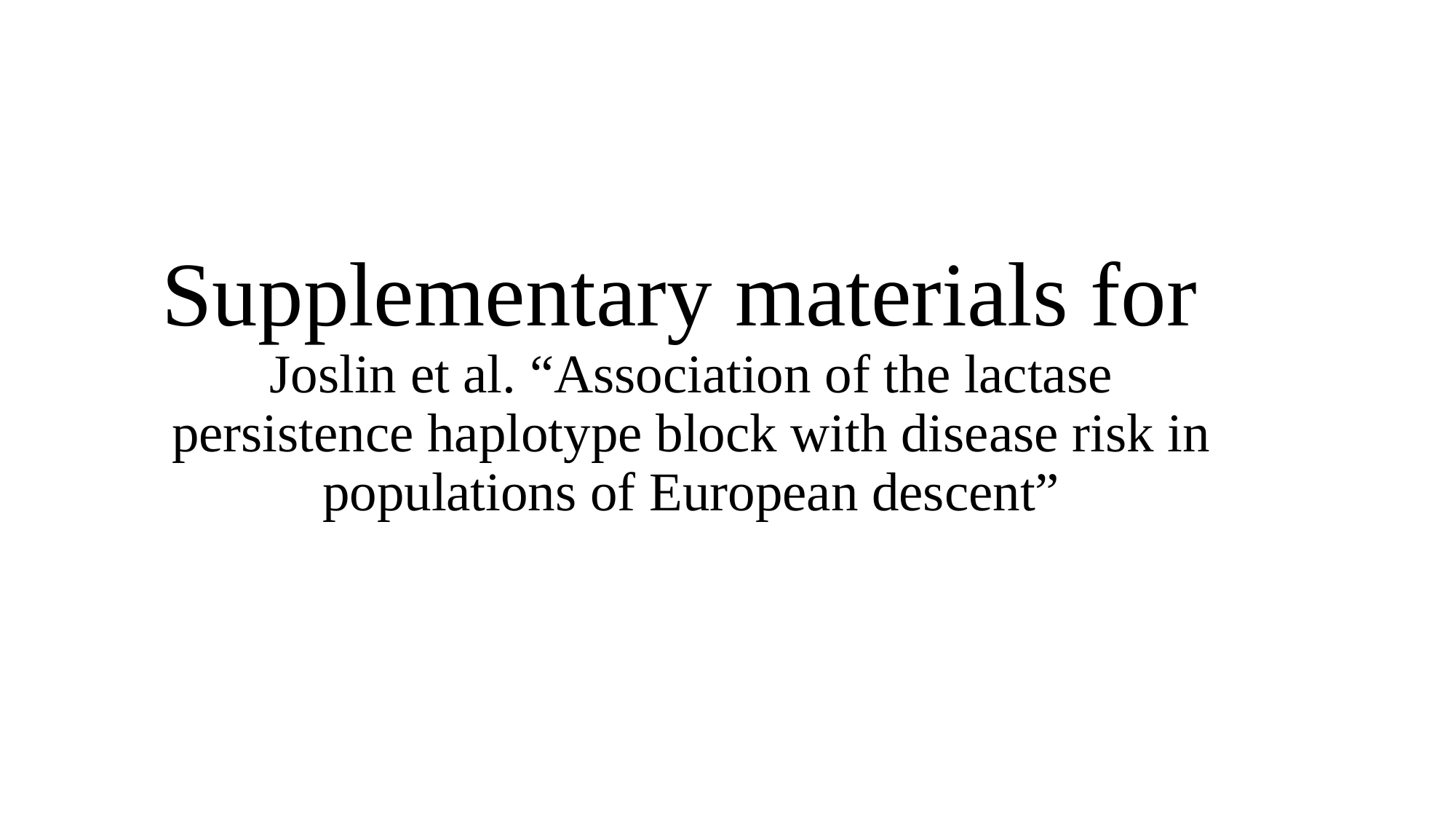

# Supplementary materials for Joslin et al. “Association of the lactase persistence haplotype block with disease risk in populations of European descent”

## Slide 2
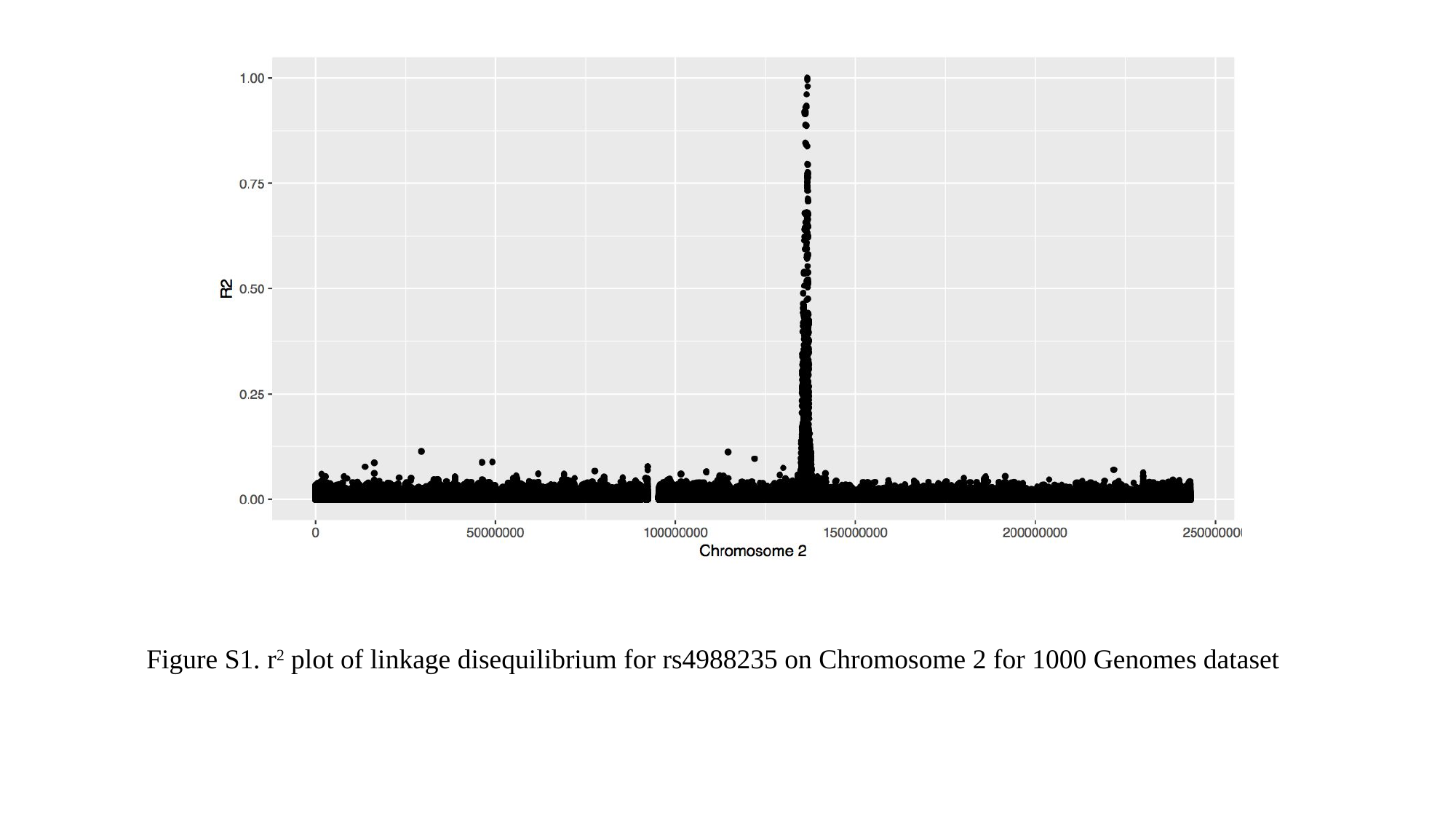

Figure S1. r2 plot of linkage disequilibrium for rs4988235 on Chromosome 2 for 1000 Genomes dataset

## Slide 3
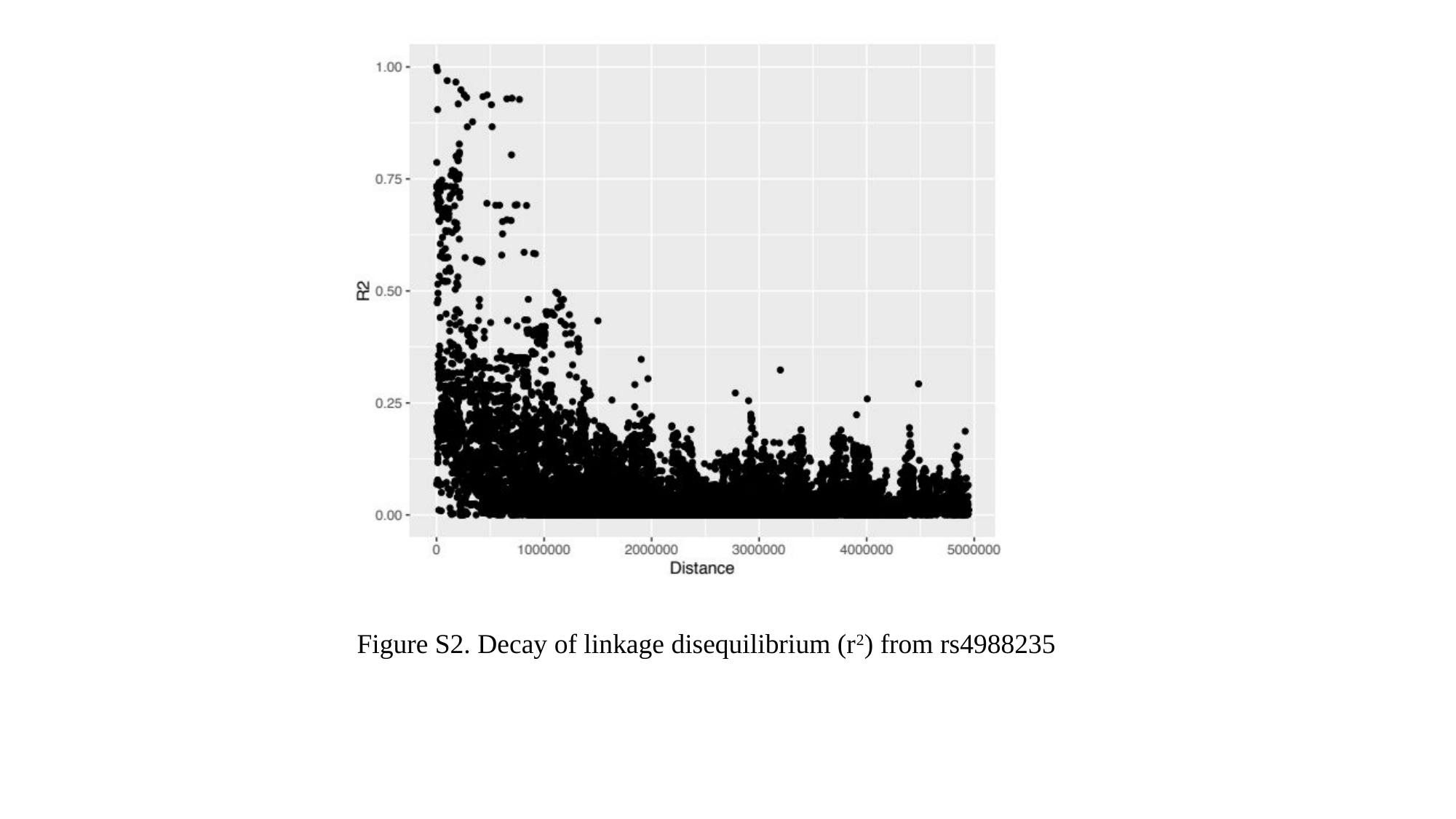

Figure S2. Decay of linkage disequilibrium (r2) from rs4988235

## Slide 4
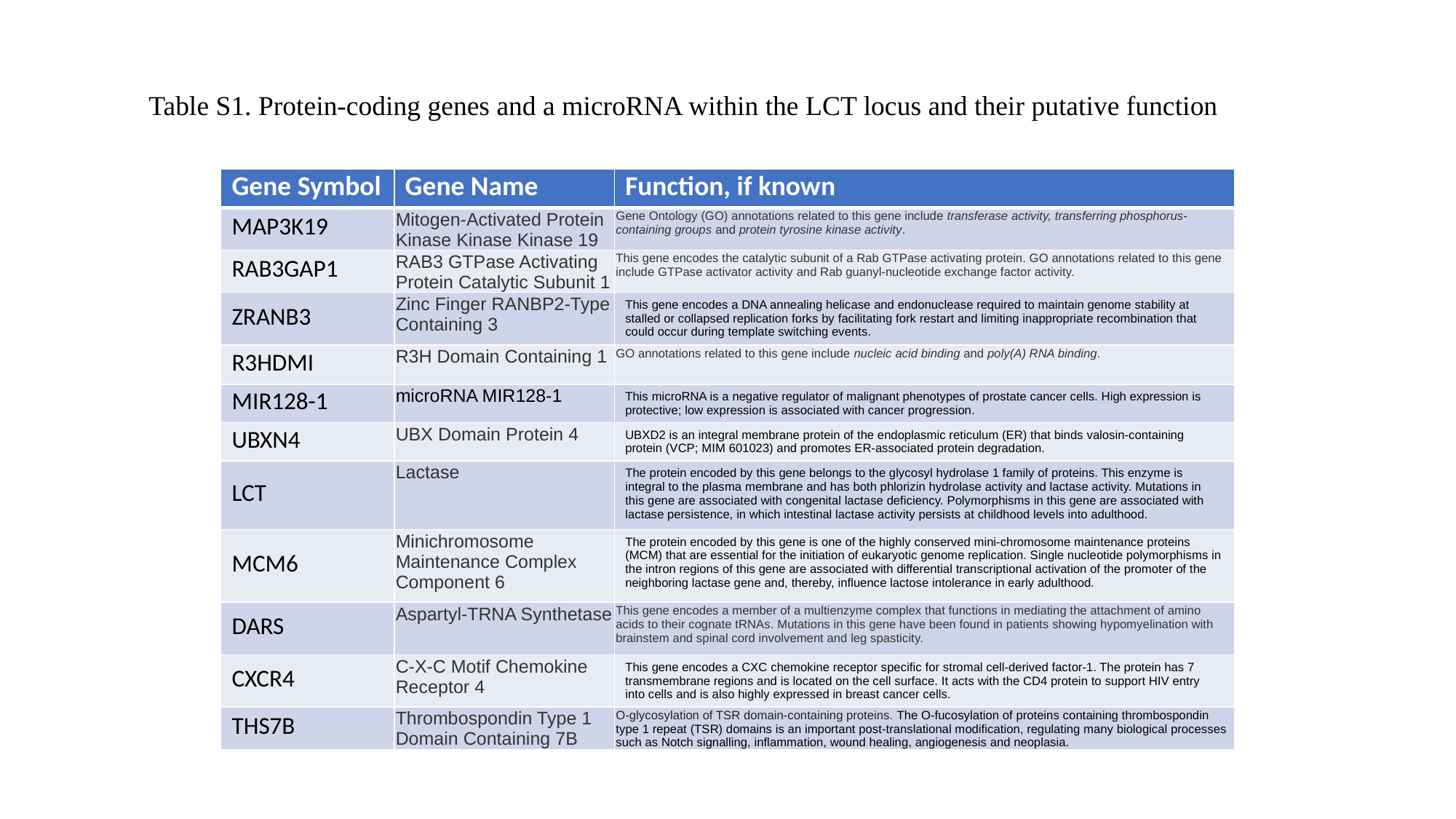

Table S1. Protein-coding genes and a microRNA within the LCT locus and their putative function
| Gene Symbol | Gene Name | Function, if known |
| --- | --- | --- |
| MAP3K19 | Mitogen-Activated Protein Kinase Kinase Kinase 19 | Gene Ontology (GO) annotations related to this gene include transferase activity, transferring phosphorus-containing groups and protein tyrosine kinase activity. |
| RAB3GAP1 | RAB3 GTPase Activating Protein Catalytic Subunit 1 | This gene encodes the catalytic subunit of a Rab GTPase activating protein. GO annotations related to this gene include GTPase activator activity and Rab guanyl-nucleotide exchange factor activity. |
| ZRANB3 | Zinc Finger RANBP2-Type Containing 3 | This gene encodes a DNA annealing helicase and endonuclease required to maintain genome stability at stalled or collapsed replication forks by facilitating fork restart and limiting inappropriate recombination that could occur during template switching events. |
| R3HDMI | R3H Domain Containing 1 | GO annotations related to this gene include nucleic acid binding and poly(A) RNA binding. |
| MIR128-1 | microRNA MIR128-1 | This microRNA is a negative regulator of malignant phenotypes of prostate cancer cells. High expression is protective; low expression is associated with cancer progression. |
| UBXN4 | UBX Domain Protein 4 | UBXD2 is an integral membrane protein of the endoplasmic reticulum (ER) that binds valosin-containing protein (VCP; MIM 601023) and promotes ER-associated protein degradation. |
| LCT | Lactase | The protein encoded by this gene belongs to the glycosyl hydrolase 1 family of proteins. This enzyme is integral to the plasma membrane and has both phlorizin hydrolase activity and lactase activity. Mutations in this gene are associated with congenital lactase deficiency. Polymorphisms in this gene are associated with lactase persistence, in which intestinal lactase activity persists at childhood levels into adulthood. |
| MCM6 | Minichromosome Maintenance Complex Component 6 | The protein encoded by this gene is one of the highly conserved mini-chromosome maintenance proteins (MCM) that are essential for the initiation of eukaryotic genome replication. Single nucleotide polymorphisms in the intron regions of this gene are associated with differential transcriptional activation of the promoter of the neighboring lactase gene and, thereby, influence lactose intolerance in early adulthood. |
| DARS | Aspartyl-TRNA Synthetase | This gene encodes a member of a multienzyme complex that functions in mediating the attachment of amino acids to their cognate tRNAs. Mutations in this gene have been found in patients showing hypomyelination with brainstem and spinal cord involvement and leg spasticity. |
| CXCR4 | C-X-C Motif Chemokine Receptor 4 | This gene encodes a CXC chemokine receptor specific for stromal cell-derived factor-1. The protein has 7 transmembrane regions and is located on the cell surface. It acts with the CD4 protein to support HIV entry into cells and is also highly expressed in breast cancer cells. |
| THS7B | Thrombospondin Type 1 Domain Containing 7B | O-glycosylation of TSR domain-containing proteins. The O-fucosylation of proteins containing thrombospondin type 1 repeat (TSR) domains is an important post-translational modification, regulating many biological processes such as Notch signalling, inflammation, wound healing, angiogenesis and neoplasia. |

## Slide 5
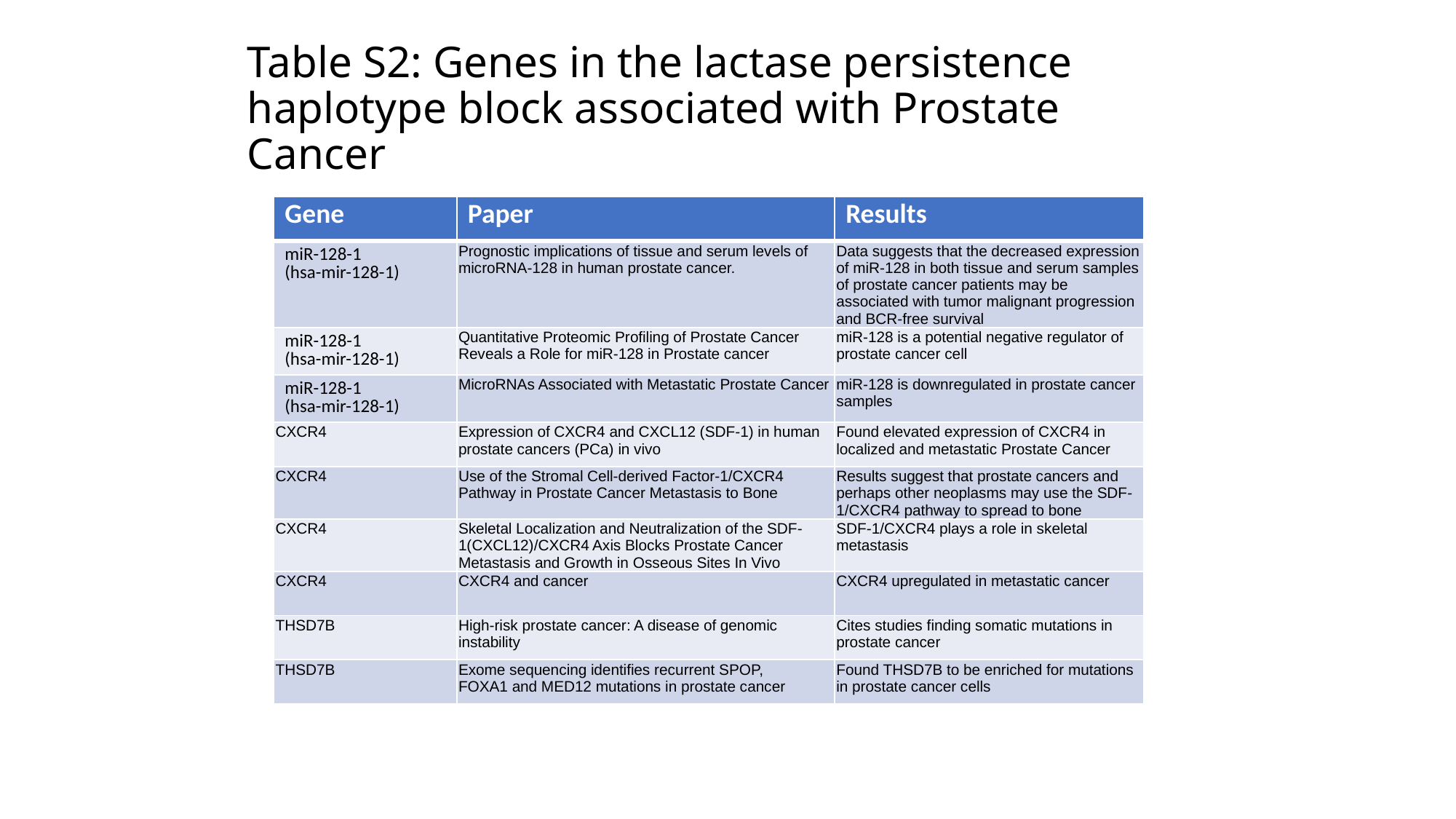

# Table S2: Genes in the lactase persistence haplotype block associated with Prostate Cancer
| Gene | Paper | Results |
| --- | --- | --- |
| miR-128-1 (hsa-mir-128-1) | Prognostic implications of tissue and serum levels of microRNA-128 in human prostate cancer. | Data suggests that the decreased expression of miR-128 in both tissue and serum samples of prostate cancer patients may be associated with tumor malignant progression and BCR-free survival |
| miR-128-1 (hsa-mir-128-1) | Quantitative Proteomic Profiling of Prostate Cancer Reveals a Role for miR-128 in Prostate cancer | miR-128 is a potential negative regulator of prostate cancer cell |
| miR-128-1 (hsa-mir-128-1) | MicroRNAs Associated with Metastatic Prostate Cancer | miR-128 is downregulated in prostate cancer samples |
| CXCR4 | Expression of CXCR4 and CXCL12 (SDF-1) in human prostate cancers (PCa) in vivo | Found elevated expression of CXCR4 in localized and metastatic Prostate Cancer |
| CXCR4 | Use of the Stromal Cell-derived Factor-1/CXCR4 Pathway in Prostate Cancer Metastasis to Bone | Results suggest that prostate cancers and perhaps other neoplasms may use the SDF-1/CXCR4 pathway to spread to bone |
| CXCR4 | Skeletal Localization and Neutralization of the SDF-1(CXCL12)/CXCR4 Axis Blocks Prostate Cancer Metastasis and Growth in Osseous Sites In Vivo | SDF-1/CXCR4 plays a role in skeletal metastasis |
| CXCR4 | CXCR4 and cancer | CXCR4 upregulated in metastatic cancer |
| THSD7B | High-risk prostate cancer: A disease of genomic instability | Cites studies finding somatic mutations in prostate cancer |
| THSD7B | Exome sequencing identifies recurrent SPOP, FOXA1 and MED12 mutations in prostate cancer | Found THSD7B to be enriched for mutations in prostate cancer cells |

## Slide 6
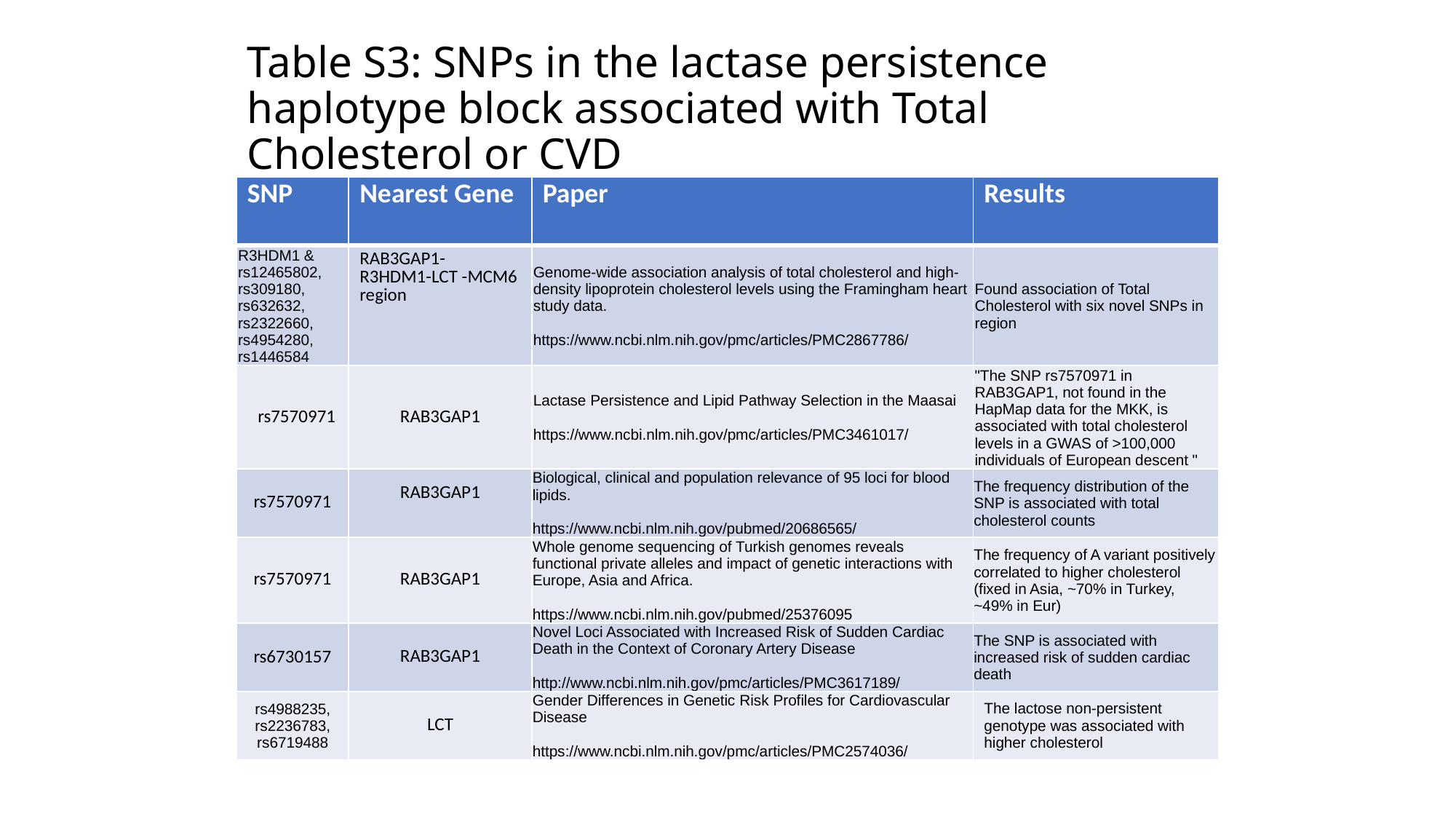

# Table S3: SNPs in the lactase persistence haplotype block associated with Total Cholesterol or CVD
| SNP | Nearest Gene | Paper | Results |
| --- | --- | --- | --- |
| R3HDM1 & rs12465802, rs309180, rs632632, rs2322660, rs4954280, rs1446584 | RAB3GAP1- R3HDM1-LCT -MCM6 region | Genome-wide association analysis of total cholesterol and high-density lipoprotein cholesterol levels using the Framingham heart study data. https://www.ncbi.nlm.nih.gov/pmc/articles/PMC2867786/ | Found association of Total Cholesterol with six novel SNPs in region |
| rs7570971 | RAB3GAP1 | Lactase Persistence and Lipid Pathway Selection in the Maasai https://www.ncbi.nlm.nih.gov/pmc/articles/PMC3461017/ | "The SNP rs7570971 in RAB3GAP1, not found in the HapMap data for the MKK, is associated with total cholesterol levels in a GWAS of >100,000 individuals of European descent " |
| rs7570971 | RAB3GAP1 | Biological, clinical and population relevance of 95 loci for blood lipids. https://www.ncbi.nlm.nih.gov/pubmed/20686565/ | The frequency distribution of the SNP is associated with total cholesterol counts |
| rs7570971 | RAB3GAP1 | Whole genome sequencing of Turkish genomes reveals functional private alleles and impact of genetic interactions with Europe, Asia and Africa. https://www.ncbi.nlm.nih.gov/pubmed/25376095 | The frequency of A variant positively correlated to higher cholesterol (fixed in Asia, ~70% in Turkey, ~49% in Eur) |
| rs6730157 | RAB3GAP1 | Novel Loci Associated with Increased Risk of Sudden Cardiac Death in the Context of Coronary Artery Disease http://www.ncbi.nlm.nih.gov/pmc/articles/PMC3617189/ | The SNP is associated with increased risk of sudden cardiac death |
| rs4988235, rs2236783, rs6719488 | LCT | Gender Differences in Genetic Risk Profiles for Cardiovascular Disease https://www.ncbi.nlm.nih.gov/pmc/articles/PMC2574036/ | The lactose non-persistent genotype was associated with higher cholesterol |

## Slide 7
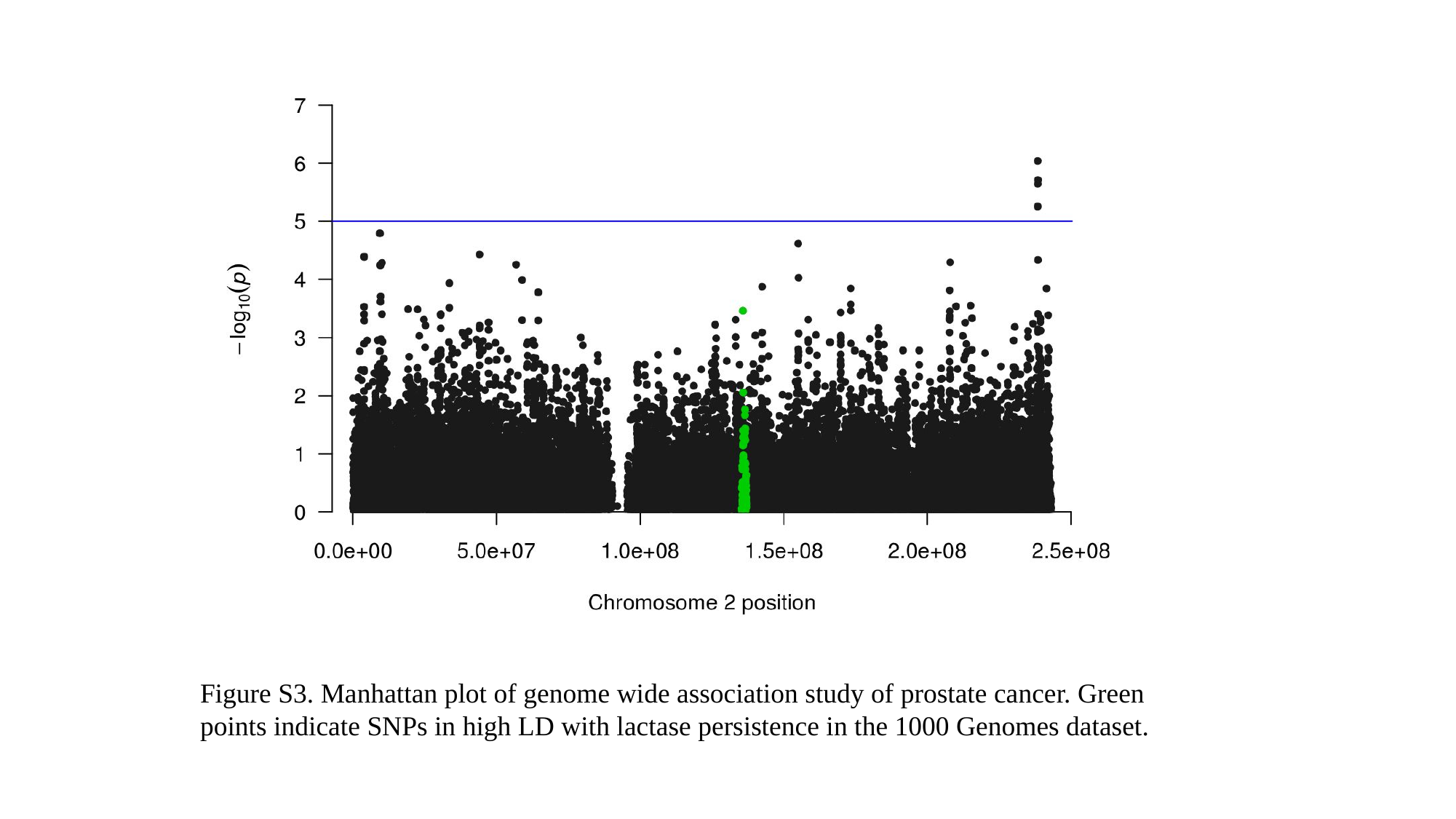

Figure S3. Manhattan plot of genome wide association study of prostate cancer. Green points indicate SNPs in high LD with lactase persistence in the 1000 Genomes dataset.

## Slide 8
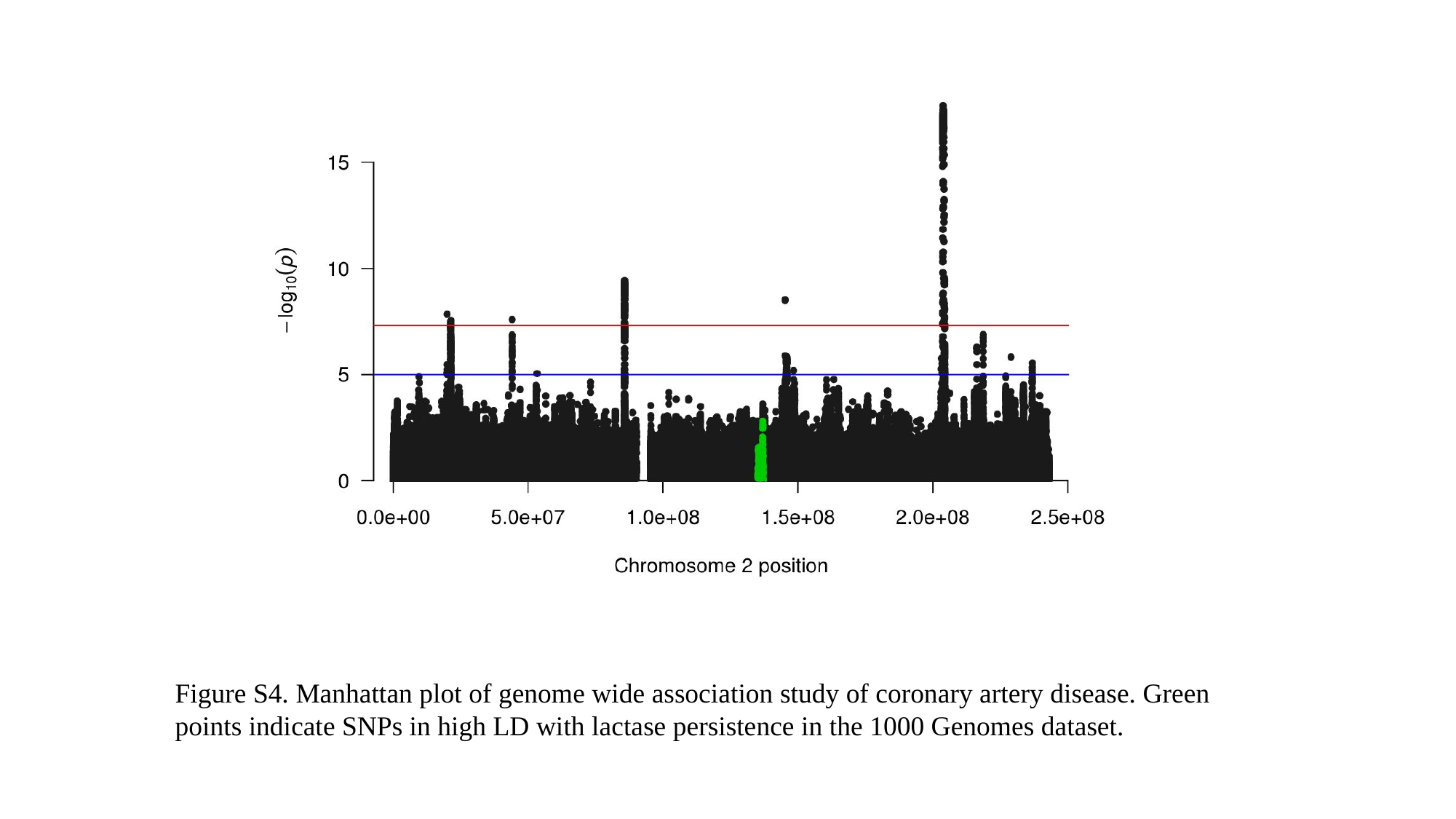

Figure S4. Manhattan plot of genome wide association study of coronary artery disease. Green points indicate SNPs in high LD with lactase persistence in the 1000 Genomes dataset.

## Slide 9
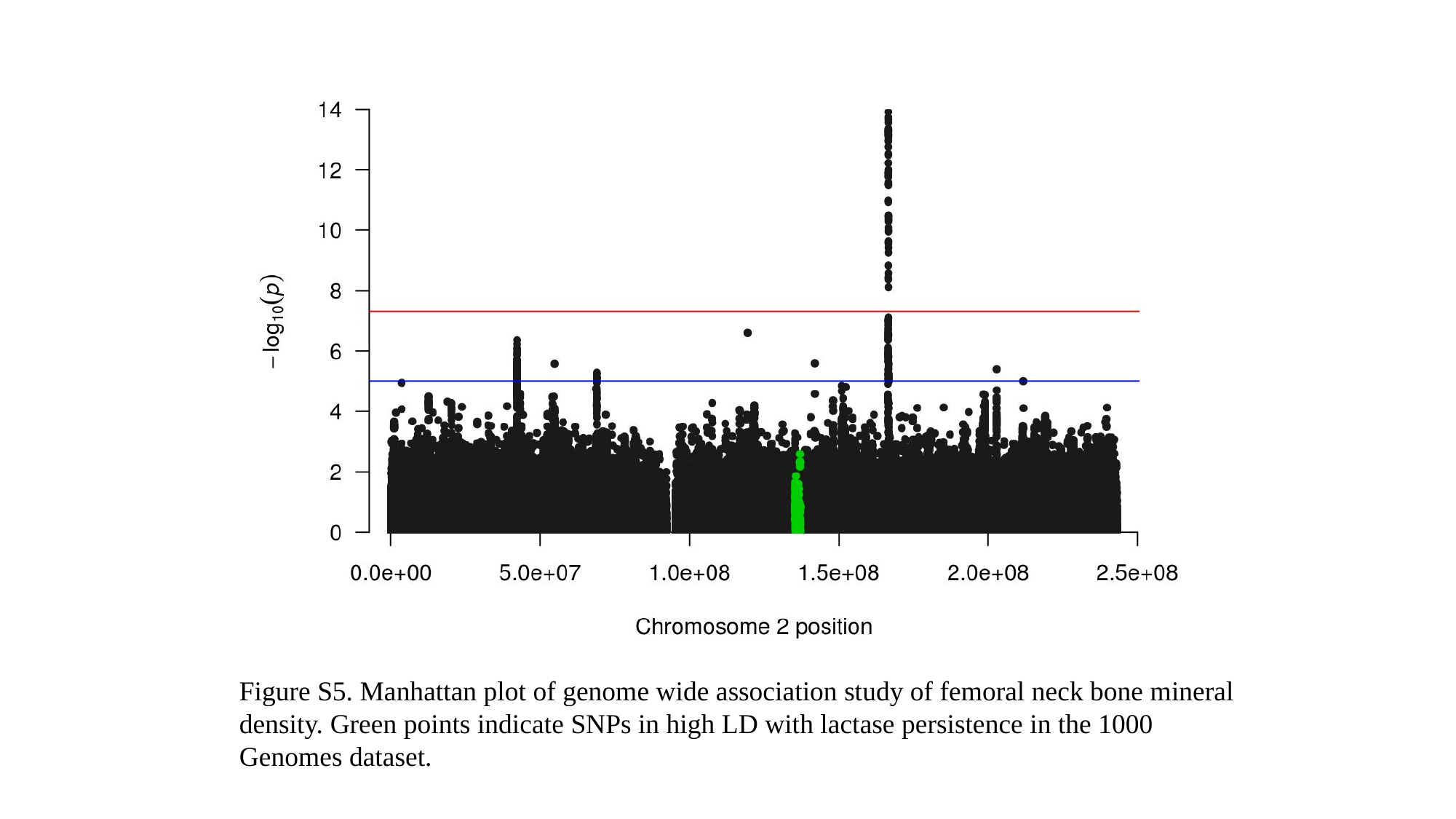

Figure S5. Manhattan plot of genome wide association study of femoral neck bone mineral density. Green points indicate SNPs in high LD with lactase persistence in the 1000 Genomes dataset.

## Slide 10
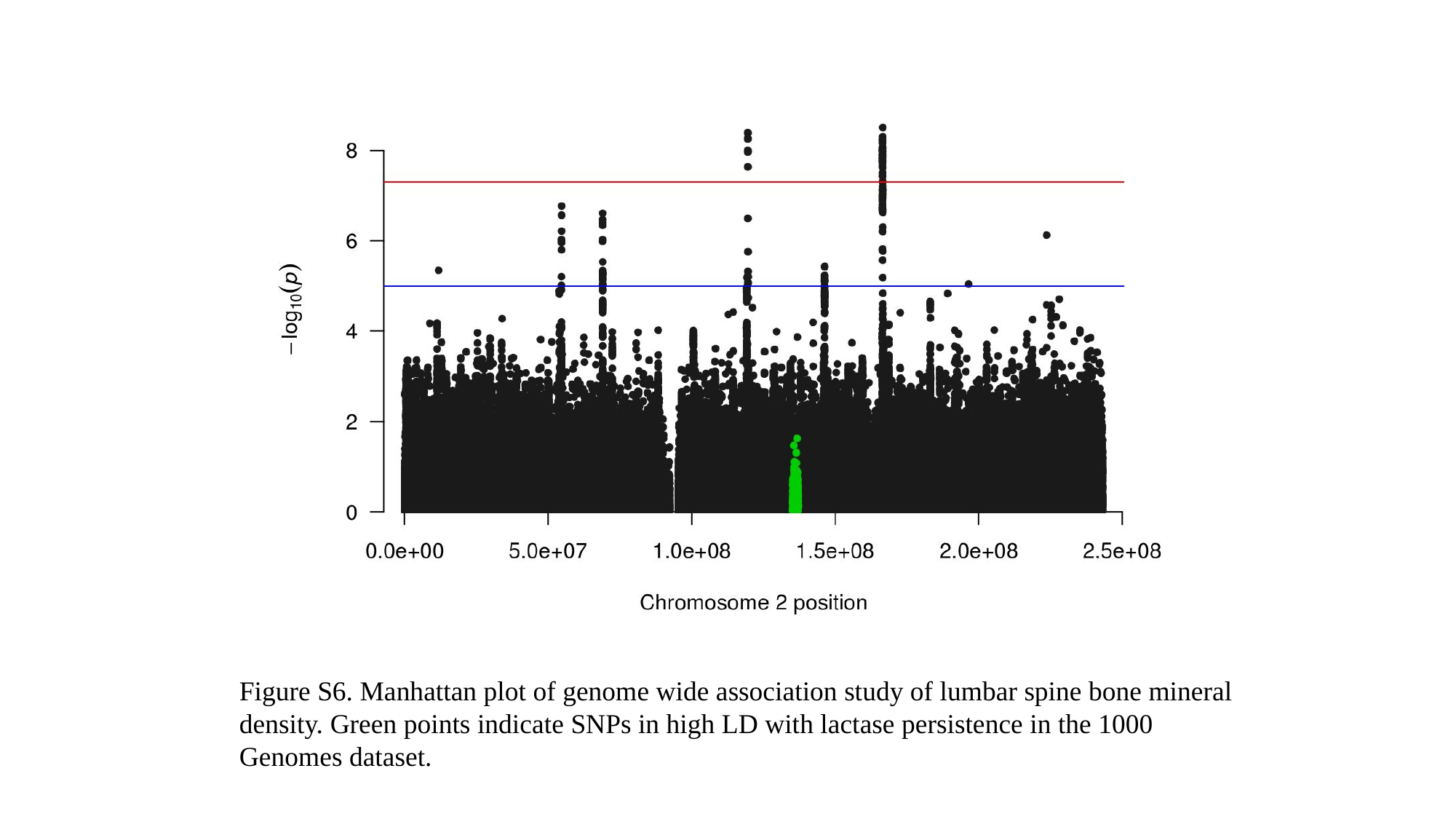

Figure S6. Manhattan plot of genome wide association study of lumbar spine bone mineral density. Green points indicate SNPs in high LD with lactase persistence in the 1000 Genomes dataset.

## Slide 11
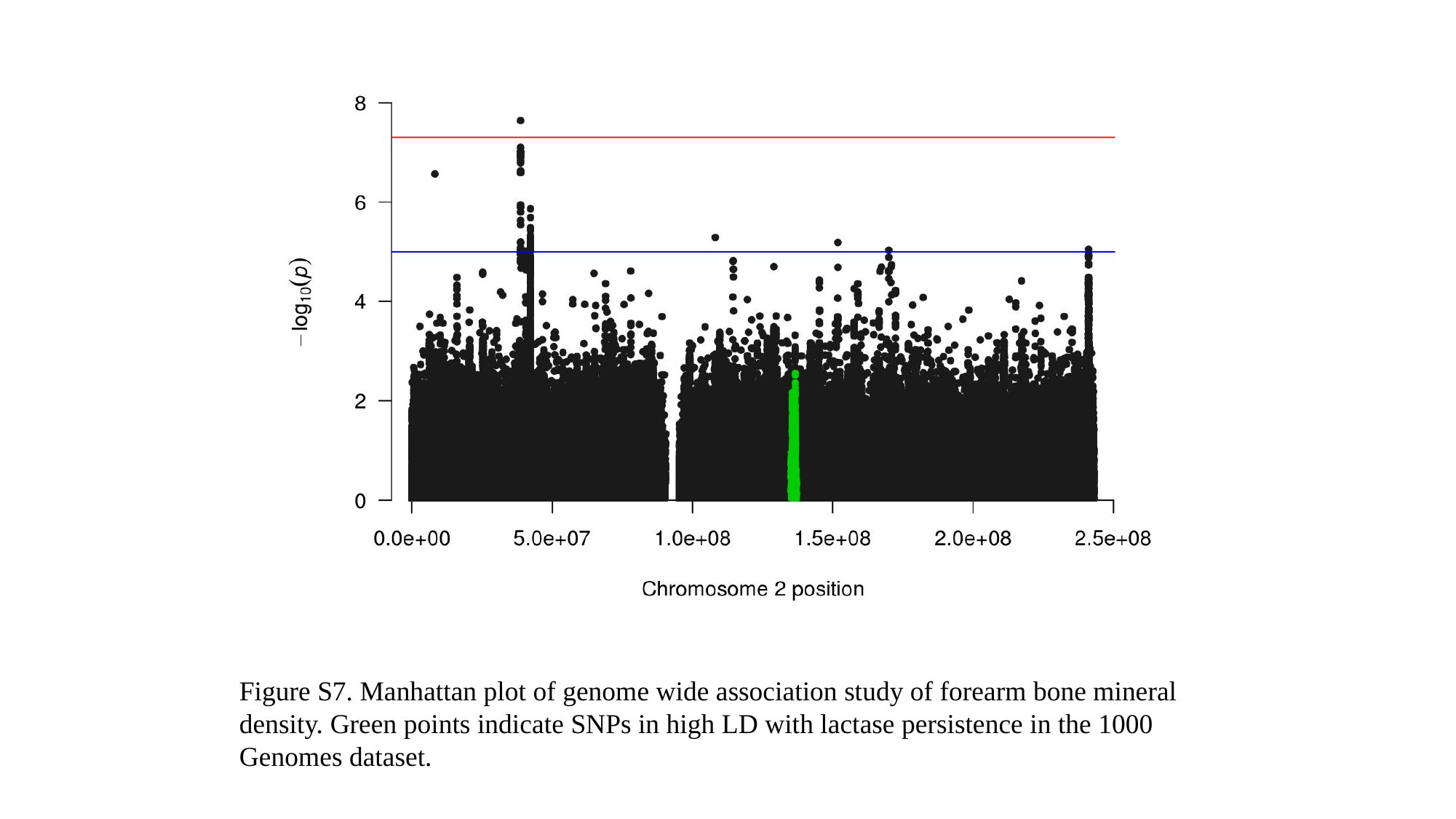

Figure S7. Manhattan plot of genome wide association study of forearm bone mineral density. Green points indicate SNPs in high LD with lactase persistence in the 1000 Genomes dataset.

## Slide 12
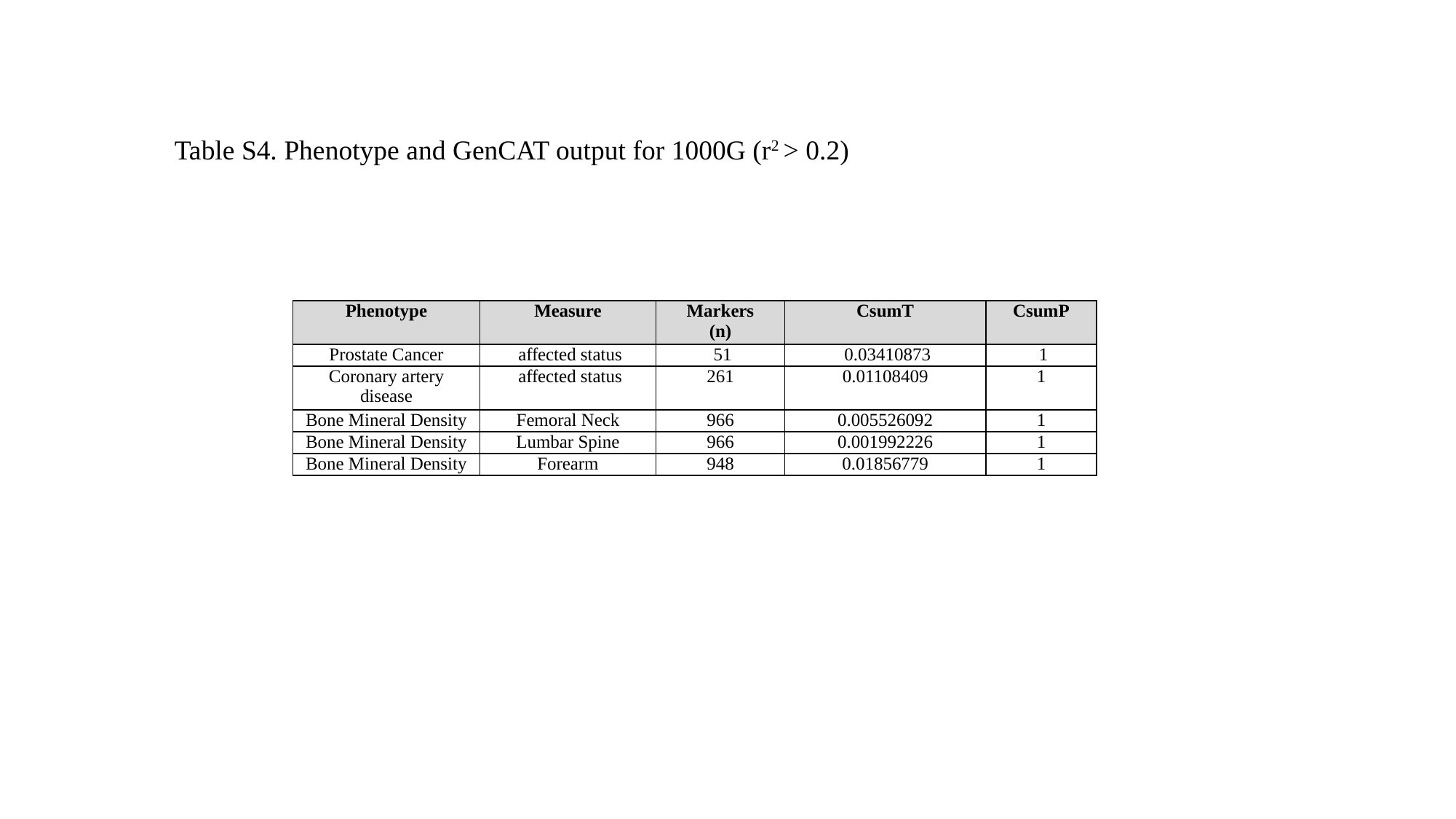

Table S4. Phenotype and GenCAT output for 1000G (r2 > 0.2)
| Phenotype | Measure | Markers (n) | CsumT | CsumP |
| --- | --- | --- | --- | --- |
| Prostate Cancer | affected status | 51 | 0.03410873 | 1 |
| Coronary artery disease | affected status | 261 | 0.01108409 | 1 |
| Bone Mineral Density | Femoral Neck | 966 | 0.005526092 | 1 |
| Bone Mineral Density | Lumbar Spine | 966 | 0.001992226 | 1 |
| Bone Mineral Density | Forearm | 948 | 0.01856779 | 1 |
